# Fluorescence-guided surgery for osteoradionecrosis of the jaw: a retrospective study

**DOI:** 10.1177/03000605221104186

**Published:** 2022-06-13

**Authors:** Suad Aljohani, Riham Fliefel, Teresa Franziska Brunner, Aristeidis Chronopoulos, Nada Binmadi, Sven Otto

**Affiliations:** 1 Department of Oral Diagnostic Sciences, Faculty of Dentistry, King Abdulaziz University, Jeddah, Saudi Arabia; 2 Department of Oral and Maxillofacial Surgery and Facial Plastic Surgery, Ludwig Maximilians University, Munich, Germany; 3 Experimental Surgery and Regenerative Medicine (ExperiMed), Department of Surgery, Ludwig Maximilians University, Munich, Germany; 4 Department of Oral and Maxillofacial Surgery, Faculty of Dentistry, Alexandria University, Alexandria, Egypt

**Keywords:** Osteoradionecrosis, osteoradionecrosis of the jaw, osteonecrosis of the jaw, fluorescence-guided surgery, radiotherapy, patient management

## Abstract

**Objective:**

Osteoradionecrosis of the jaw (ORNJ) is one of the most severe head and neck complications in patients treated with radiotherapy. The goal of treatment is to suppress ORNJ progression. Currently, surgical removal of necrotic bone is an effective management approach for advanced stages. In this study, we present our experience in managing ORNJ using fluorescence-guided surgery.

**Methods:**

Nineteen ORNJ lesions in 15 hospitalized patients were treated with fluorescence-guided surgery. We retrospectively reviewed patients’ demographic data, comorbidities, local preceding event, location, ORNJ stage, and treatment outcomes with a median follow-up of 12 months.

**Results:**

Twelve lesions (63%) were treated surgically under tetracycline fluorescence, and seven lesions (37%) were surgically treated under auto-fluorescence. Overall, four lesions (21%) achieved complete mucosal healing, eight lesions (42%) showed partial mucosal healing with bone exposure and no signs or symptoms of inflammation, and seven lesions (37%) were progressive. The results showed that either healing or ORNJ stabilization was achieved in 63% of lesions (n = 12).

**Conclusion:**

Fluorescence-guided surgery can be beneficial in curing or stabilizing ORNJ. However, randomized clinical trials are needed to confirm these findings.

## Introduction

Current advancements in the management of head and neck cancer offer a remarkable prognosis and can achieve high survival rates. Radiotherapy (RT) combined with surgery and chemotherapy has become effective in case management. Whereas the prognosis is remarkably improved, it comes with some limitations. For example, osteoradionecrosis of the jaws (ORNJ) is a severe adverse effect of craniofacial RT. The evolution of RT, better clinical implementation, and prevention strategies have significantly decreased the incidence of ORNJ from 37.5% several decades ago to less than 5% presently.^1–3^ Most cases appear within 3 years after RT, with a median of 13 months between RT and ORNJ.^2,4^ ORNJ affects the mandible more than the maxilla owing to the greater vascularity and lower density of the maxillary medullary bone.

Despite the large body of literature focusing on ORNJ, there is no consensus among scholars regarding its definition. The most widely accepted definition of ORNJ is based on clinical presentation: irradiated jaw bone exposed through the overlying mucosa or skin without healing for at least 3 months in patients with a history of RT for the head and/or neck without malignancy recurrence at the affected site.^5–8^ ORNJ occurs spontaneously or is triggered by local infection, denture-related trauma, and extraction.^9,10^ Thus, careful dental evaluation and treatment of oral infection or trauma before RT can reduce the risk of ORNJ.

Surgical removal of necrotic bone is challenging because preserving as much bone as possible is crucial to avoid jaw fracture or persistent mandibular bone loss. At the same time, necrotic bone must be completely removed to lower the risk of relapse. Many surgeons use bone bleeding as an indicator of vital bone despite unreliable evidence.^11^ Numerous imaging techniques can be used to effectively estimate the extent of necrotic bone. However, these methods cannot be used as a guide for bone excision as they lack sensitivity and specificity.^12,13^ In 2009, Pautke et al. introduced fluorescence-guided bone excision for the treatment of medication-related osteonecrosis of the jaw (MRONJ).^14,15^ The technique was prospectively investigated among 15 patients with 20 MRONJ lesions, with an 85% healing rate after a 4-week follow-up.^16^ Several studies have also found fluorescence-guided bone excision to be an effective tool in discriminating between viable and necrotic bone, thereby aiding in more preserved yet complete bone removal.^17–20^ Another study validated the ability of the fluorescence-guided surgical technique to differentiate between vital and necrotic bone based on the results of histopathological analysis of fluorescent and non-fluorescent bone.^11^ An interesting finding was that histological evidence of bone necrosis was detected for clinically vital bone with normal color, texture, and bleeding, which failed to display fluorescence under a fluorescence illumination lamp. Thus, fluorescence guidance during necrotic bone removal is more accurate than relying on bone color, texture, and bleeding.

Ristow and Pautke reported that vital bone can demonstrate fluorescence (auto-fluorescence) using the VELscope® System (LED Dental, White Rock, BC, Canada) without prior administration of tetracycline.^21^ The authors suggested the use of auto-fluorescence instead of tetracycline fluorescence for detection of necrotic bone. Several studies have reported the same observation regarding auto-fluorescence of viable bone without tetracycline labeling.^22–24^ Recent investigations have used a mini-pig model to compare the two techniques and confirmed the lack of any macroscopic or histological difference.^25^

Given that fluorescence-guided surgery offers good results in patients with MRONJ in terms of the healing rate and ease of use, in this study, we report our experience in auto-fluorescence and tetracycline fluorescence for ORNJ. We also aimed to investigate the correlation between healing and patient-related variables, tumor-related variables, comorbidities, and ORNJ-related variables.

## Methods

### Study design

We conducted a retrospective, single-center study among patients with biopsy-proven ORNJ who were treated with fluorescence-guided surgery between February 2012 and March 2018 at the Department of Oral and Maxillofacial Surgery, Ludwig Maximilians University, Munich. ORNJ was clinically defined as the presence of exposed necrotic bone in the jawbones, irradiated with no history of antiresorptive medications or metastasis to the affected site. Ethical approval was obtained from Ludwig Maximilians University Research Ethics Committee (19-610). Informed consent was obtained from all individual participants. The reporting of this study conforms to the Strengthening the Reporting of Observational Studies in Epidemiology (STROBE) guidelines.^26^

The inclusion criteria were a diagnosis of ORNJ in patients treated with RT alone or in combination with surgery and/or chemotherapy, persistent bone exposure for 3 months or more, treatment of ORNJ using fluorescence-guided surgery, histologically proven ORNJ, and a follow-up period of 6 months or more. Exclusion criteria were a history of antiresorptive treatment before, during, or after RT; evidence of recurrent malignancy of the jaws; and a follow-up period of less than 6 months.

### Diagnostics

The diagnosis of ORNJ was established based on clinical and radiological findings. ORNJ lesions were classified into three stages according to the Notani et al. classification (Table 1).^27^

**Table 1.** Staging system used to classify ORNJ lesions in this study.

Staging system	Stages
Notani et al.^26^	Stage I: ORNJ limited to the alveolar bone
	Stage II: ORNJ limited to the alveolar bone and/or the mandible above the level of the mandibular alveolar canal
	Stage III: ORNJ that extends to the mandible below the level of the mandibular alveolar canal and lesions and/or skin fistula and/or pathologic fracture

ORNJ, Osteoradionecrosis of the jaw.

### Outcomes

At the final follow-up visit, the treatment outcomes were recorded and divided into three categories: completely healed, not healed but stable (with no signs or symptoms of infection), and progressive lesions.

### Data analysis

We collected the following patient data: demographic data, sites of malignancy and clinical stage, radiation dose, systemic comorbidities, preceding oral events, ORNJ stage and site, surgical treatment, and outcomes. We then conducted descriptive data assessment. In the present study, the primary outcome was mucosal ORNJ healing in the absence of ORNJ-related signs and symptoms, including pain, exposed bone, intra- or extra-oral fistula, and pathologic fracture. We investigated the correlation between independent and dependent variables in the analysis. The independent variables were age, sex, tumor site and stage, radiation dose, systemic comorbidities, ORNJ-related variables as mentioned above, and the fluorescence technique. The dependent variable was mucosal healing of ORNJ after a fluorescence-guided surgical procedure. Variables were analyzed using IBM SPSS Statistics v. 22 (IBM Corp., Armonk, NY, USA). We used the chi-square test, Student *t*-test, and Kruskal–Wallis test for the analysis. The significance level was set at *p* = 0.05.

## Results

### Patients

Fifteen consecutive patients with 19 lesions were included in the study, 12 (80%) men and 3 (20%) women, with a mean patient age of 64 ± 10 years (range, 51 to 78 years). Table 2 presents the sites and stages of primary tumors and their associated comorbidities. The mean period between the first radiation dose and ORNJ diagnosis was 33 ± 28.5 months (range, 3 to 89 months). The mean radiation dose was 62.7 ± 7.4 Gy (range, 50 to 70 Gy).

**Table 2.** Initial tumor characteristics and comorbidities.

Variable	Category	Number of patients (percentage)
Malignancy	Tongue	3 (20%)
	Pharynx	3 (20%)
	Tongue and floor of the mouth	2 (13.3%)
	Palate	1 (6.7%)
	Floor of the mouth	2 (13.3%)
	Skin	1 (6.7%)
	Tonsils	1 (6.7%)
	Alveolar process	1 (6.7%)
	Thyroid	1 (6.7%)
Tumor stage	1	3 (15.8%)
	2	4 (21.1%)
	3	6 (40%)
	4	2 (13.3%)
Comorbidities	Diabetes mellitus	3 (20%)
	Cardiovascular disease	9 (60%)
	Smoking	9 (60%)
	Alcohol	8 (53.3%)
	Chemotherapy	9 (47.4%)
	Corticosteroids	0 (0%)

Approximately half of the lesions occurred with no associated dental event or pathology (n = 8, 42%). However, four lesions were preceded by tooth extraction (21%), in which one of the associated denture pressure points was reported. Marginal and periapical periodontitis was observed at the ORNJ site in three lesions (n = 4, 21%); however, only marginal periodontitis was identified in two lesions (n = 2, 10.5%). A remaining root was found in one case (n = 1, 5%).

All lesions were located in the mandible (89.5%) except for two lesions in the maxilla (10.5%). The lesions sites are summarized in Table 3. Regarding ORNJ stage, we observed 6 stage I lesions (31.6%), 10 stage II lesions (52.6%), and 3 stage III lesions (15.8%).

**Table 3.** Sites of ORNJ.

Region	Number of lesions (percentage)
Molar area	6 (31.6%)
Premolar area	4 (21%)
Premolar and molar area	3 (15.8%)
Anterior area	1 (5.3%)
Anterior area extending to premolar area	2 (10.5%)
Anterior area extending to posterior teeth area	2 (10.5%)
Whole alveolar process	1 (5.3%)

ORNJ, Osteoradionecrosis of the jaw.

Panoramic radiographs and computed tomography scans were conducted for all patients to determine the extent of ORNJ. Biopsies were taken from all lesions to rule out malignancy.

### Surgical treatment

Fluorescence-guided surgery with tetracycline bone labeling was performed in the first 10 patients (first 12 lesions). Patients received 100 mg of doxycycline twice a day for 7 to 10 days preoperatively. After surgery, the patients were given intravenous ampicillin/sulbactam (2 gm/1 gm) three times daily or clindamycin, 1800-mg dose daily, in case of allergy to penicillin; the dosage was continued for 3 to 4 days (until hospital discharge).

Auto-fluorescence was performed for the remaining five patients (seven lesions). These patients did not receive doxycycline but were given the second intravenous course of antibiotics described above, at least 1 day preoperatively. All patients were switched to oral antibiotics for 10 days after hospital discharge (amoxicillin/clavulanic acid, 875 mg/125 mg three times daily or clindamycin, 600 mg three times daily, for patients allergic to penicillin).

**Figure 1.**
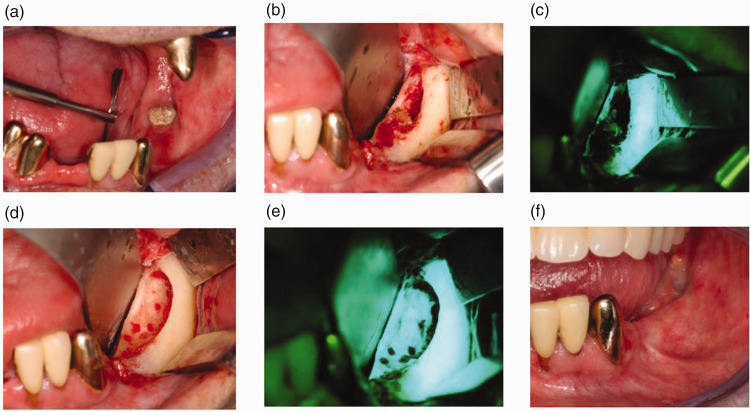
(a) A 63-year-old male patient presented with exposed necrotic bone in his left mandible. Medical history was significant for head and neck radiotherapy owing to pharyngeal carcinoma. (b) Intraoperative image after elevation of periosteal flap. (c) Fluorescence view before necrotic bone removal; dull green fluorescence evident at the area of necrosis. (d) and (e) After necrotic bone removal and smoothening of sharp bone edges; bright homogenous green-fluorescent bone was observed and (f) Intraoral image 6 months after surgery with complete mucosal healing.

All patients were operated under general anesthesia. All ORNJ lesions were treated using fluorescence-guided surgery. First, the mucoperiosteal flap was elevated. After that, fluorescence (with the VELscope® System) was used to distinguish necrotic bone, as detailed by Otto et al.^16,18^ Bone with dull or no fluorescence was gradually removed until brightly fluorescent bone was evident (Figure 1). Any teeth within the necrotic bone were extracted. After the removal of necrotic bone, sharp bone edges were smoothed, followed by tension-free watertight primary closure of the mucoperiosteal flaps (Serafit 3-0, SERAG-Wiesner GmbH, Germany).

### Treatment outcomes

The median follow-up period was 14.8 ± 9.7 months (range, 6 to 37 months). Four lesions (21%) were resolved; eight lesions (42%) showed partial mucosal healing in the absence of clinical or radiological progression with no ORNJ-related signs and symptoms. Four lesions (21%) recurred with progression, and three lesions (16%) recurred and were complicated by loss of mandibular continuity.

Of the 12 lesions treated using fluorescence-guided surgery with tetracycline bone labeling, 16.7% were healed (n = 2). By comparison, 28.6% of seven lesions surgically treated with auto-fluorescence guidance demonstrated complete mucosal healing (n = 2). Table 4 provides outcomes of the fluorescence technique and the ORNJ initial stage.

**Table 4.** Outcomes in relation to the stage and fluorescence technique.

Stage	Fluorescence technique	Outcome			
		Resolved	Stable	Progressive with no loss of mandibular continuity	Progressive with loss of mandibular continuity
I	Tetracycline fluorescence	2	1	0	0
	Auto-fluorescence	2	1	0	0
II	Tetracycline fluorescence	0	4	1	2
	Auto-fluorescence	0	0	3	0
III	Tetracycline fluorescence	0	1	0	1
	Auto-fluorescence	0	1	0	0

The stage of ORNJ was inversely associated with healing (*p* = 0.004). However, no association was found between healing and sex, type of malignancy, tumor stage, diabetes mellitus, cardiovascular disease, smoking, alcohol, chemotherapy, tetracycline labeling, site of the lesion within the dental arch, suppuration, pain, the period between RT and ORNJ onset, and dose of radiation.

## Discussion

ORNJ management remains controversial with no evidence-based guidelines. Management ranges from non-surgical treatment to surgical excision to large resections. Regardless of the modality, ORNJ treatment is challenging, with a limited success rate, which may lead to non-healing wounds, progressive lesions, loss of continuity defects, and large resections. Many studies have advocated non-surgical measures yet to be validated by high-level clinical evidence.^28,29^ Annane et al. conducted a multicenter randomized, placebo-controlled, double-blind trial of the ORN96 Study Group and found worse outcomes in the hyperbaric oxygen arm;^30^ thus, the trial was stopped. A recent systematic review evaluated pentoxifylline–tocopherol or pentoxifylline–tocopherol–clodronate for ORNJ management and concluded that randomized controlled clinical trials were crucial to draw evidence-based conclusions about their efficacy.^30^ Because necrotic bone can never be revitalized, surgical resection is a reasonable management approach, particularly for advanced ORNJ stages. A study conducted among a diverse cohort of 116 patients with ORNJ confirmed that radical resection of necrotic bone was a valuable treatment owing to the positive clinical outcomes.^31^

Early lesion management could prevent ORNJ progression and offer a better treatment response. Thus, surgical treatment combined with antibiotic therapy is crucial even for early ORNJ stages. As reported in other studies, advanced ORNJ stages have a poorer cure rate after surgical treatment.^3,27^ In the present study, a significant association was observed between ORNJ stage and healing (*p* = 0.004). Accordingly, the healing rate in our study for stage I lesions was higher than that of stage II and III lesions. Among the six stage I lesions in our study, 66.7% of lesions (n = 4) were resolved versus 0% for stage II and III ORNJ. However, two (33.3%) lesions persisted in the absence of any ORNJ-related signs and symptoms (stable). Thus, ORNJ treatment remains challenging, with a limited success rate, and might require several surgical interventions owing to the impaired repair capacity of irradiated bone.^5^ On the basis of this consideration, the treatment objective is to prevent ORNJ progression and improve patients’ quality of life. It is worth noting that the ORNJ management strategy should be selected with the individual patient’s status in mind.

Fluorescence imaging has been used to detect resection margins of the necrotic bone secondary to MRONJ.^14–17^ A prospective cohort study including 20 patients with MRONJ who underwent fluorescence-guided surgery reported complete mucosal healing in all but one patient over a follow-up of 18 months.^19^ This technique was based on tetracycline derivatives that showed fluorescence properties under excitation light. Tetracycline has a high affinity for calcium and can accumulate during active bone remodeling. Thus, vital bone exhibits bright green fluorescence under the VELscope® System whereas necrotic bone emits no or dull fluorescence. Afterward, successful auto-fluorescence-guided necrotic bone removal (without prior intake of tetracycline), verified by histopathological investigation, was found to have a good rate of healing.^21^ A randomized clinical trial demonstrated the healing rate after fluorescence-guided bone surgery with and without tetracycline,^32^ with healing observed in 89% of the tetracycline fluorescence group and 94% of the auto-fluorescence group. A recent study reported the absence of macroscopic and microscopic differences between tetracycline-induced fluorescence and auto-fluorescence in both viable and necrotic bone.^25^ Similarities between the two techniques are attributed to auto-fluorescence of collagen and cell-filled bone lacunae.

In the present study, 12 lesions (63%) were treated using tetracycline fluorescence-guided surgery and auto-fluorescence-guided surgery was used in 7 lesions (37%). Two lesions in each group demonstrated complete mucosal healing in the absence of relapse-related signs and symptoms (16.7% and 28.6%, respectively). Moreover, ORNJ stabilization was achieved in 50% and 28.6% of the tetracycline-fluorescence group and auto-fluorescence group, respectively (Table 4). The aforementioned healing rates were for the first surgical intervention, which is not usually successful owing to the progressive nature of ORNJ. Thus, it is common to carry out several revision surgeries in ORNJ treatment. Notani et al. reported that the cure rate after the first surgery was significantly lower than that after the second surgery, with 50% and 86.7%, respectively.^27^ In the present study, the first surgical intervention using fluorescence guidance resulted in healed or stabilized ORNJ in 63% of lesions.

ORNJ is more progressive than MRONJ, with a higher rate of complications such as pathologic fractures and extra-oral fistulae.^33,34^ The periosteal blood supply is more affected in ORNJ than MRONJ, probably explaining the worse ORNJ treatment outcomes.^35^ A recent study reported a complete mucosal healing rate of 81.7% (67 of 82 lesions) after fluorescence-guided bone removal in patients with MRONJ.^20^ However, this rate was only 21% in the present study. From our experience and the results of several studies conducted at our institute, the outcomes of fluorescence-guided surgery for ORNJ are worse than those for MRONJ.^16,18,20^ This is because ORNJ is a more severe type of bone necrosis that could be associated with hypoxia, hypocellularity, and hypovascularity as direct effects of RT.^7^

Numerous factors contribute to the risk of ORNJ. Total radiation dose, smoking, alcohol consumption, local oral factors including poor oral hygiene, periodontitis, mucosal trauma, and extraction have all been linked to an increased risk of ORNJ.^9^ A radiation dose of more than 65 Gy has been reported to predispose the patient to ORNJ.^36^ In line with that report, the mean radiation dose in the present study was 62.7 ± 7.4 Gy. ORNJ has been frequently linked to dental extraction after RT.^1^ In a multicenter retrospective study of 392 patients, periapical periodontitis and tooth extraction after RT were found to be significant independent risk factors for ORNJ development.^37^ On the contrary, in a case-control study of 1023 patients who underwent RT for oral cavity cancer and oropharyngeal cancer, 44 patients developed ORNJ, with no associated dental events in 83% of them.^9^ In the present study, ORNJ occurred without a prior local event or surgical intervention in approximately half of lesions (n = 8, 42%). However, extraction and periodontitis were identified in 21% (n = 4) and 11% (n = 2) of lesions, respectively.

## Conclusion

ORNJ remains a challenging and severe complication of RT. This study was the first to investigate the use of autofluorescence-guided surgery in ORNJ. The goal of management is mucosal healing or at least prevention of ORNJ progression, aiming to control pain and improve patients’ quality of life. Despite the inherent limitations of the current study owing to its retrospective nature and small sample size, we demonstrated that fluorescence-guided surgery is a valuable intraoperative tool that can facilitate the identification of necrotic bone and offer reliable and accurate guidance during bone excision. Randomized clinical trials are needed to evaluate this tool for ORNJ management.

## Supplemental Material

sj-pdf-1-imr-10.1177_03000605221104186 - Supplemental material for Fluorescence-guided surgery for osteoradionecrosis of the jaw: a retrospective study Click here for additional data file. Supplemental material, sj-pdf-1-imr-10.1177_03000605221104186 for Fluorescence-guided surgery for osteoradionecrosis of the jaw: a retrospective study by Suad Aljohani, Riham Fliefel, Teresa Franziska Brunner, Aristeidis Chronopoulos, Nada Binmadi and Sven Otto in Journal of International Medical Research

## Data Availability

The datasets analyzed in this study are available from the corresponding author upon request.
